# Comparison of screening for gestational diabetes mellitus by oral glucose tolerance tests done in the non-fasting (random) and fasting states

**DOI:** 10.1007/s00592-014-0660-5

**Published:** 2014-10-15

**Authors:** Viswanathan Mohan, Manni Mohanraj Mahalakshmi, Balaji Bhavadharini, Kumar Maheswari, Gunasekaran Kalaiyarasi, Ranjit Mohan Anjana, Ram Uma, Sriram Usha, Mohan Deepa, Ranjit Unnikrishnan, Sonak D. Pastakia, Belma Malanda, Anne Belton, Arivudainambi Kayal

**Affiliations:** 1 WHO Collaborating Centre for Non Communicable Diseases Prevention and Control and IDF Centre of Education, Madras Diabetes Research Foundation & Dr. Mohan’s Diabetes Specialities Centre, 4, Conran Smith Road, Gopalapuram, Chennai, 600 086 India; 2 Seethapathy Clinic and Hospital, Chennai, India; 3 Associates in Clinical Endocrinology Education and Research (ACEER), Chennai, India; 4 College of Pharmacy, Purdue University, West Lafayette, IN USA; 5 International Diabetes Federation, Brussels, Belgium

**Keywords:** Gestational diabetes mellitus, Fasting OGTT, Non-fasting OGTT, Diabetes in Pregnancy Study Group of India (DIPSI), WHO 1999 criteria, IADPSG, Screening

## Abstract

**Aim:**

The Diabetes in Pregnancy Study Group of India (DIPSI) guidelines recommend the non-fasting 75-g oral glucose tolerance test (OGTT) as a single-step screening and diagnostic test for gestational diabetes mellitus (GDM). The aim of this study was to compare the DIPSI criteria with the World Health Organization (WHO) 1999 and the International Association of Diabetes and Pregnancy Study Groups (IADPSG) criteria for GDM.

**Methods:**

A total of 1,031 pregnant women attending antenatal clinics in urban and rural Tamil Nadu, India, underwent a 75-g OGTT in both non-fasting and fasting states, 2–3 days apart. Venous plasma glucose was measured using an autoanalyser, and GDM was diagnosed by DIPSI, WHO 1999 and IADPSG criteria.

**Results:**

Of the 83 women identified to have GDM by WHO 1999 criteria, only 23 were diagnosed by DIPSI criteria. Of the 106 women diagnosed to have GDM by the IADPSG criteria, only 24 were diagnosed by DIPSI. The DIPSI non-fasting OGTT 2-h VPG cut point of 140 mg/dl (7.8 mmol/l) had a very low sensitivity when compared to the WHO 1999 criteria (sensitivity 27.7 %, specificity 97.7 %) and IADPSG criteria (sensitivity 22.6 %, specificity 97.8 %).

**Conclusions:**

The DIPSI non-fasting OGTT criteria cannot be recommended for diagnosis of GDM due to its low sensitivity. Thus, as a single-step diagnostic test for GDM, the fasting OGTT needs to be done. When this is not possible, the well-established two-step procedure using the 50-g glucose challenge test as an initial screening test, followed by the diagnostic fasting OGTT, can be continued.

## Introduction

Gestational diabetes mellitus (GDM) is defined as carbohydrate intolerance of any severity, first recognized during pregnancy [1]. GDM is associated with considerably increased rates of maternal and perinatal complications. The prevalence of GDM varies widely based on the diagnostic criteria used and the ethnic group studied [2]. Unfortunately, there is no international consensus on the screening and diagnostic criteria for GDM.

In 1999, the World Health Organization (WHO) introduced criteria for diagnosis of GDM on the basis of a 2-h venous plasma glucose (VPG) cut-off value of 140 mg/dl (7.8 mmol/l), after the administration of 75 g of glucose [3]. The WHO 1999 criteria have become popular, particularly in developing countries, because it is simpler than the two-step procedure [4]. In 2010, based on the Hyperglycemia and Adverse Pregnancy Outcomes (HAPO) study, the International Association of Diabetes and Pregnancy Study Groups (IADPSG) proposed a new set of criteria which has since been adopted in many countries [5, 6]. Recently, the WHO has also adopted the IADPSG criteria [7].

## Methods

As part of the Women in India with GDM Strategy (WINGS) programme [8], this study was undertaken to evaluate the non-fasting OGTT to evaluate the Diabetes in Pregnancy Study Group of India (DIPSI) non-fasting criteria [9]. The aim was to compare the sensitivity and specificity of the non-fasting OGTT with the WHO 1999 and IADPSG criteria for the diagnosis of GDM, in order to assess whether this non-fasting OGTT could be recommended for use in resource-constrained settings where a fasting OGTT may be difficult to do in all pregnant women.

This is a cross-sectional study carried out on consecutive pregnant women attending urban antenatal care centres in Chennai City as well as rural primary health centres in Kanchipuram District in Tamil Nadu State in southern India. The study was conducted between January and November 2013. A standardized questionnaire was used to collect details including demography, family history of diabetes and the obstetric history. Height was measured using a stadiometer (SECA Model 213, Seca Gmbh Co, Hamburg, Germany) to the nearest 0.1 cm, and weight was measured with an electronic weighing machine (SECA Model 803, Seca Gmbh Co) to the nearest 0.1 kg. The body mass index (BMI) was calculated using the formula weight (in kg) divided by height in meters (squared). All procedures followed were in accordance with the ethical standards and in keeping with the Declaration of Helsinki 1975, as revised in 2008. Permission was obtained from the Director of Public Health and the Health Secretary, Government of Tamil Nadu, to undertake the WINGS programme. All participants gave written informed consent prior to participating in the study. The study was approved by the Institutional Ethics Committee of the Madras Diabetes Research Foundation, Chennai, India.

Most women in India report to antenatal clinics in the non-fasting state. Accordingly, a total of 1,400 consecutive pregnant women attending 20 urban and rural clinics in Tamil Nadu in South India underwent a non-fasting OGTT using a 82.5 g oral glucose load (equivalent to 75 g of anhydrous glucose) which was administered irrespective of the timing of the last meal. A venous blood sample was drawn 2 h after the glucose was administered. All 1,400 women were then invited to return 2 or 3 days later to repeat a 75-g OGTT, this time after an overnight fast of at least 8 h. Venous samples were drawn at fasting, 1 and 2 h after the glucose load.

Blood samples were collected in sodium fluoride/Na2 EDTA vacutainer tubes to prevent glycolysis. Samples were transported to the central laboratory within 1 h in cool boxes which had gel packs to maintain the temperature between 2 and 8 °C. Plasma glucose was measured using an autoanalyser AU2700 (Beckman, Fullerton, CA), and glycated haemoglobin (HbA1c) was measured by high-performance liquid chromatography (HPLC) using variant machine (Bio-Rad, Hercules, CA). The HbA1c method is NGSP-certified. The intra- and inter-assay coefficients of variation (CV) for the glucose and HbA1c were 0.78, 1.68, 0.59 and 1.97 %, respectively. The laboratory is certified by the College of American Pathologists (CAP), USA, and the National Accreditation Board for Testing and Calibration Laboratories (NABL), Government of India.

### Definitions of GDM used in this study

1. According to the WHO 1999 criteria, diagnosis was based on a 2-h VPG value of ≥140 mg/dl (7.8 mmol/l) done in the fasting state, and this is called ‘WHO 1999 criteria’ for the purpose of this paper [3].
2. According to the IADPSG criteria, diagnosis of GDM was based on any one of the following criteria, i.e. fasting ≥92 mg/dl (5.1 mmol/l), 1 h ≥ 180 mg/dl (10 mmol/l) and 2 h ≥ 153 mg/dl (8.5 mmol/l) in the fasting state, and this is referred to as ‘IADPSG criteria’ for the purpose of this paper [5].
3. According to the DIPSI criteria, diagnosis of GDM was based on a 2-h VPG ≥140 mg/dl (7.8 mmol/l) in the non-fasting OGTT, and this is called ‘DIPSI criteria’ for the purpose of this paper [9].

### Statistical analysis

All values are expressed as the mean ± SD. Statistical analysis was performed using SPSS software (version 20) and MedCalc (version 12.7.0). Receiver operating characteristic curves were plotted using sensitivity and 1-specificity for different non-fasting 2-h VPG values against the WHO 1999 and the IADPSG criteria which were used as the gold standard, and the C statistic was calculated.

## Results

**Fig. 1:**
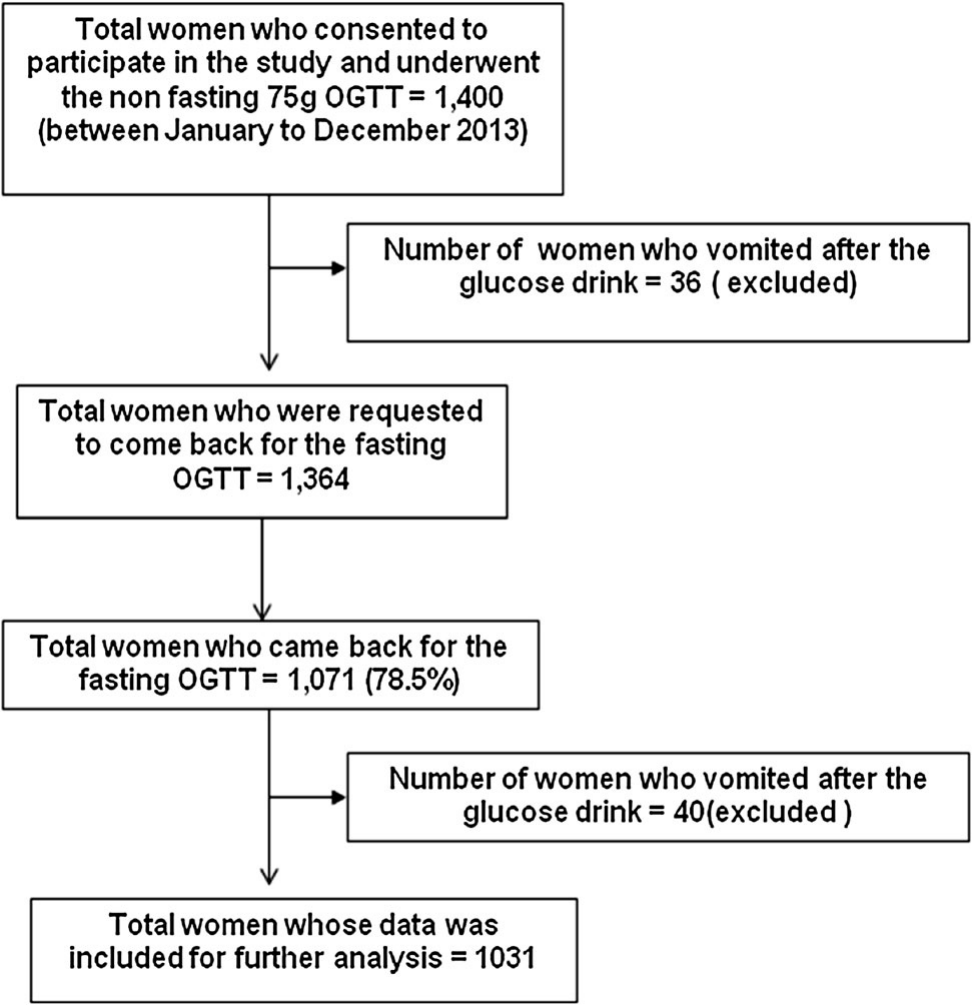
Flowchart of study procedures

A total of 1,400 pregnant women underwent the initial non-fasting OGTT. Thirty-six (2.6 %) women vomited after consuming the glucose, and they were excluded from further analysis. The remaining 1,364 women were requested to come back 2–3 days later, for the fasting OGTT, of whom 1,071 (78.5 %) came back for the test. Of these 1,071 women, 40 (3.7 %) vomited after consuming the glucose and they were excluded from further analyses **(**Fig. 1 **)**. There was no significant difference in the number of women who vomited during the fasting and non-fasting OGTT (*p* = 0.12).

The data of the remaining 1,031 women were analysed further in this study. The mean age of the 1,031 women was 24 ± 3.1 years (median 24, interquartile range 22–26 years), mean BMI 22.6 ± 4 kg/m^2^ (median 22.2, interquartile range 19.7–24.9) and mean gestational age, 23.7 ± 7.6 weeks (median 24, interquartile range 20–29.5). One hundred and twenty-eight (12.4 %) women were tested in the first trimester, 517 (50.7 %) in the second trimester and 386 (37.4 %) in the third trimester of pregnancy.

Eighty-three women (8.0 %) were diagnosed to have GDM using the WHO 1999 criteria, whereas 44 (4.2 %) women were diagnosed to have GDM using the DIPSI criteria. There were no differences in age (24.9 ± 3.1 vs. 25 ± 3 years), duration of gestation (21.9 ± 8.1 vs. 23.2 ± 6.6 weeks), BMI (24.1 ± 4.8 vs. 25.3 ± 5.1 kg/m^2^), glycated haemoglobin [5.3 ± 0.8 % (34 mmol/mol) vs. 5.4 ± 1.1 % (36 mmol/mol)] or first-degree family history of diabetes (26.5 % vs. 29.5 %) between the women diagnosed with GDM using the WHO 1999 or DIPSI criteria.

**Fig. 2:**
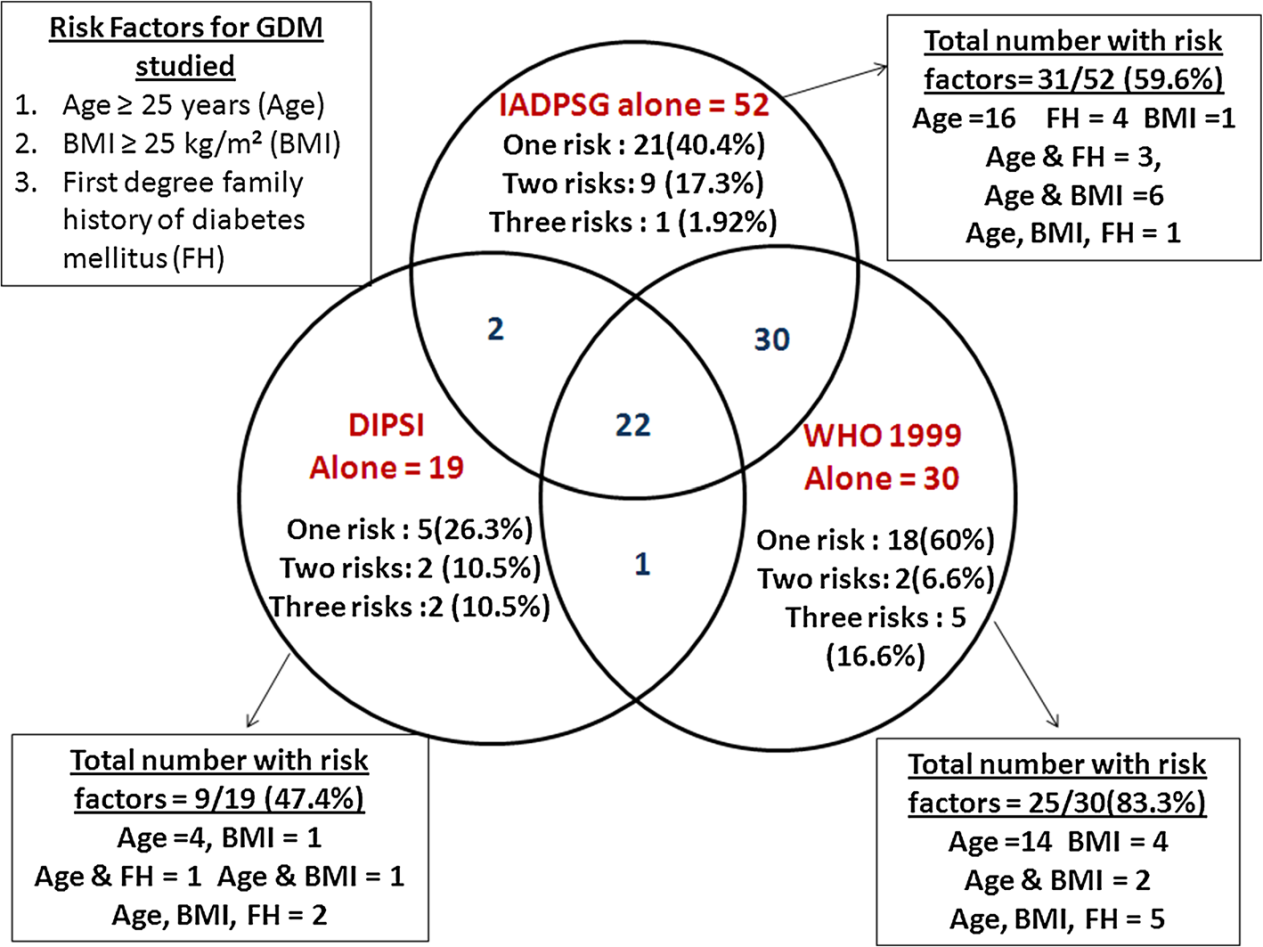
Venn diagram showing different aggregation of GDM risk factors in women diagnosed as having GDM by all three criteria

Figure 2 shows the comparison of diagnosis of GDM between WHO 1999 criteria, DIPSI non-fasting criteria and IADPSG criteria. Of the 83 women identified to have GDM by the WHO 1999 criteria, only 23 (27.7 %) women were diagnosed by the DIPSI non-fasting criteria and 52 (62.6 %) by IADPSG criteria. Conversely, of the 44 women diagnosed to have GDM by the DIPSI non-fasting criteria, only 23 (52.2 %) cases were diagnosed by the WHO 1999 criteria and 24 (54.5 %) by IADPSG criteria. Of the 106 women (10.3 %) diagnosed to have GDM by the IADPSG criteria only, 24 women (22.6 %) were diagnosed by the DIPSI non-fasting criteria and 52 (49.1 %) by WHO 1999 criteria. Only 22 women were identified by all the three criteria.

Out of 1,031 women screened, 520 women (50.4 %) had one or more risk factors for GDM (age ≥ 25 years, BMI ≥ 25 kg/m^2^, with the first-degree family history of diabetes). Of these 520, 363 (35.2 %) had only one risk factor, 134 (13 %) had two risk factors and 23 (2.2 %) had all the three risk factors for GDM. Comparison of the aggregation of GDM risk factors in women identified by the three criteria is presented in Fig. 2.

**Fig. 3:**
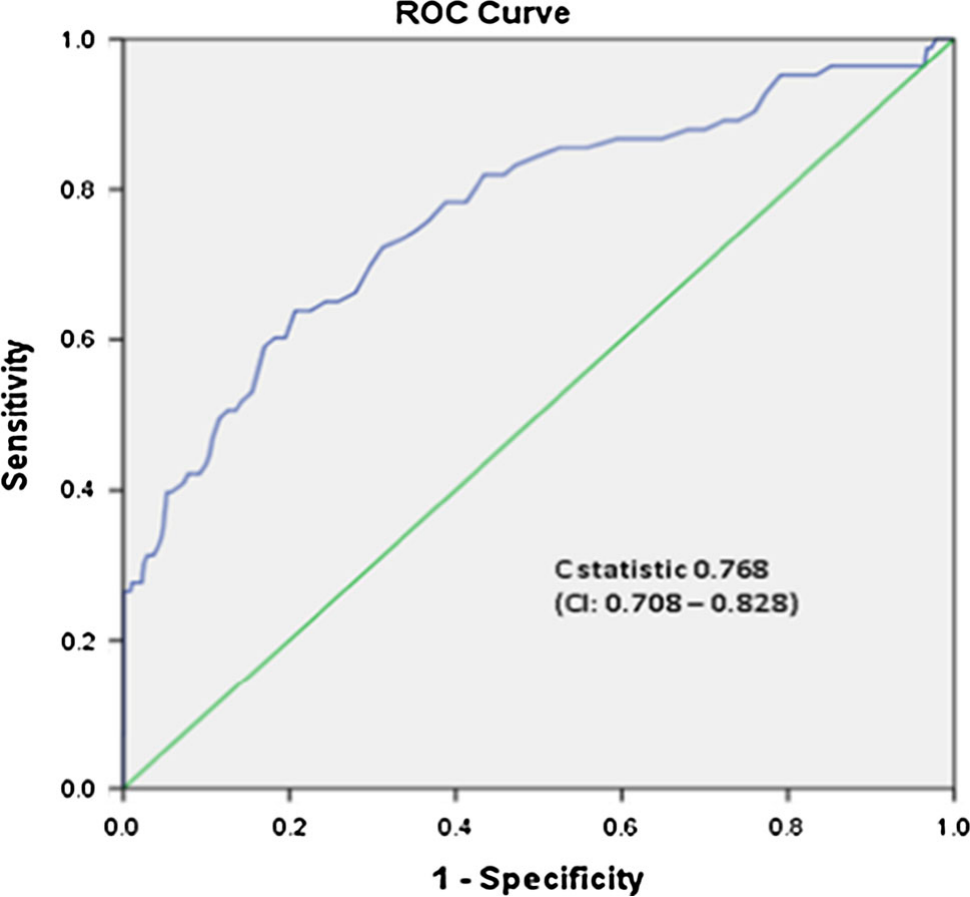
Receiver operating curve (ROC) of non-fasting 2-h venous plasma glucose versus WHO 1999 criteria

Table 1 shows that in comparison with the WHO 1999 criteria, the sensitivity of the DIPSI criteria (i.e. using the 140 mg/dl cut point) was 27.7 %, while the specificity was 97.7 % with a C statistic of 0.768 (95 % confidence interval (CI) 0.708–0.828; Fig. 3). We then looked at different non-fasting 2-h VPG cut points to see whether the sensitivity could be improved. When the non-fasting 2-h VPG value was lowered to 110 mg/dl (6.1 mmol/l), the sensitivity improved to 72.3 % (specificity 68.6 %), and at 100 mg/dl (5.5 mmol/l), it improved to 85.5 % (specificity 47.7 %).

**Table 1:** Comparison of sensitivity and specificity of different non-fasting 2-h VPG cut points in comparison with the WHO 1999 criteria for GDM

Non-fasting 2-h VPG cut point (mg/dl)	Sensitivity (%)	Specificity (%)	Positive predictive value (%)	Negative predictive value (%)	Accuracy (%)	% of population who have glucose level above this value
90 (5.0 mmol/l)	92.7	22.6	1.2	0.32	77.6	78.5
100 (5.5 mmol/l)	85.5	47.7	12.5	97.4	68.5	55.1
110 (6.1 mmol/l)	72.3	68.6	16.9	96.6	69.8	34.5
120 (6.7 mmol/l)	53.0	84.4	23	95.4	78.5	18.5
130 (7.2 mmol/l)	40.9	92.7	33	94.7	87.5	10.0
140 (7.8 mmol/l)	27.7	97.7	52.2	93.9	94.7	4.2
150 (8.3 mmol/l)	26.5	99.6	88	93.9	97.8	2.4

**Fig. 4:**
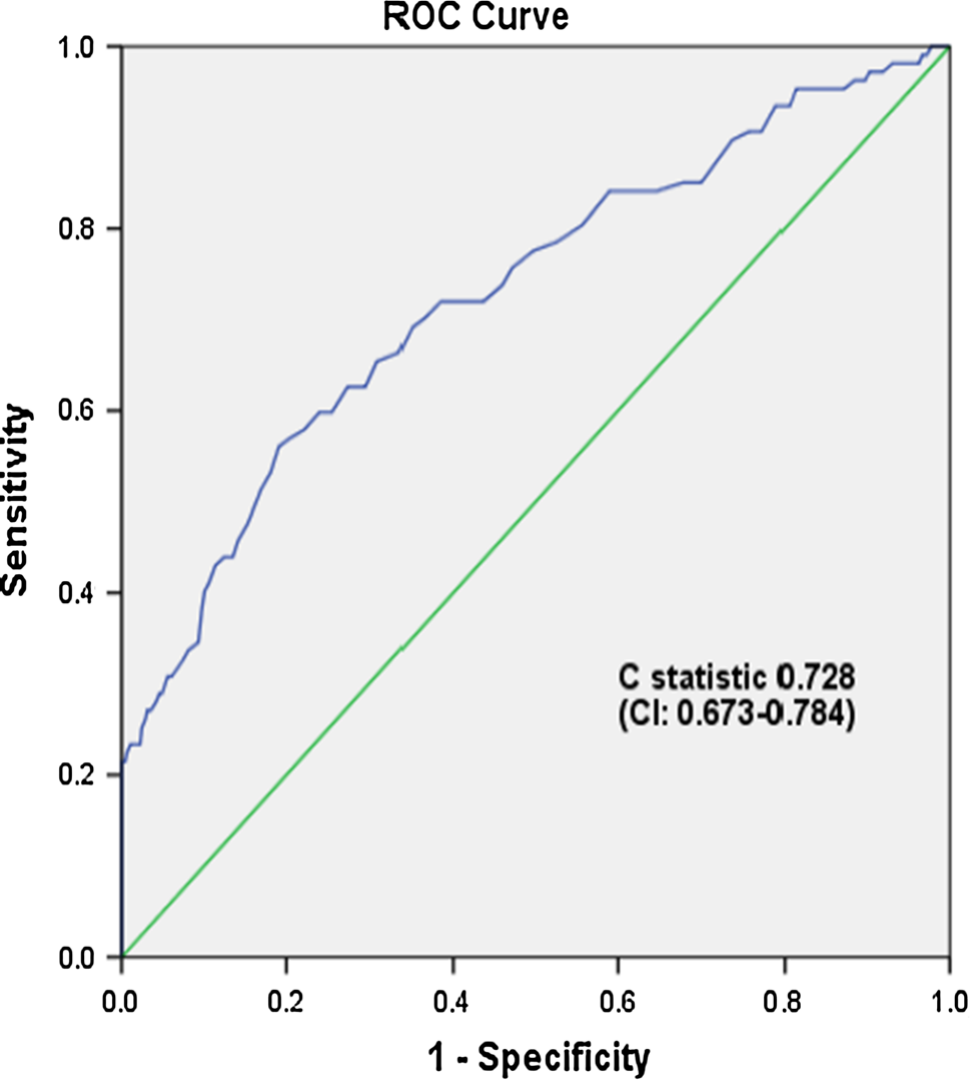
Receiver operating curve (ROC) of non-fasting 2-h venous plasma glucose versus IADPSG criteria

Table 2 shows that in comparison with the IADPSG criteria, the sensitivity of the DIPSI criteria was 22.6 %, while the specificity was 97.8 % with a C statistic of 0.728 (95 % CI 0.673–0.784; Fig. 4). When the non-fasting 2-h VPG cut point was lowered to 110 mg/dl (6.1 mmol/l) and 100 mg/dl (5.5 mmol/l), the sensitivity improved to 65.1 % (specificity 69 %) and 78.3 % (specificity 47.5 %), respectively.

**Table 2:** Comparison of sensitivity and specificity of different non-fasting 2-h VPG cut points in comparison with the IADPSG criteria for GDM

Non-fasting 2-h VPG cut point (mg/dl)	Sensitivity (%)	Specificity (%)	Positive predictive value (%)	Negative predictive value (%)	Accuracy (%)	% of population who have glucose level above this value
90 (5.0 mmol/l)	90.6	22.8	11.9	95.5	76.0	78.5
100 (5.5 mmol/l)	78.3	47.5	14.6	95.0	64.5	55.1
110 (6.1 mmol/l)	65.1	69.0	19.4	94.5	67.6	34.5
120 (6.7 mmol/l)	47.2	84.8	26.2	93.3	77.8	18.5
130 (7.2 mmol/l)	32.1	92.5	33.0	92.2	86.5	10.0
140 (7.8 mmol/l)	22.6	97.8	54.5	91.7	94.6	4.2
150 (8.3 mmol/l)	21.7	99.8	92.0	91.7	97.9	2.4

Tables 1 and 2 show that 34.5–55.1 % of women had non-fasting 2-h VPG value ≥110 mg/dl (6.1 mmol/l) and ≥100 mg/dl (5.5 mmol/l), respectively.

## Discussion

The Diabetes in Pregnancy Study Group of India (DIPSI) has laid down guidelines for diagnosis of GDM and proposes that the OGTT can be performed in a non-fasting state. The DIPSI guidelines further suggest that a non-fasting OGTT using a 2-h VPG value of 140 mg/dl (7.8 mmol/l) can be used as a *single-step, definitive, screening and diagnostic test for GDM.* These guidelines were based on a single-centre study from southern India which reported 100 % sensitivity and 100 % specificity for this cut point compared to the WHO 1999 criteria which also uses the same cut point of 140 mg/dl (7.8 mmol/l) [9]. The DIPSI guidelines (2009) have since been widely accepted and are being used all over India [10–12].

As part of the International Diabetes Federation (IDF) sponsored WINGS programme, we wanted to determine the most feasible screening test for GDM in resource-constrained parts of the developing world. Accordingly, one of the aims of WINGS was to look at the feasibility of adopting the DIPSI non-fasting OGTT and this was compared to the WHO 1999 and IADPSG criteria for diagnosis of GDM, both of which use fasting OGTTs. This study shows that the non-fasting OGTT has poor sensitivity compared to both the WHO 1999 criteria (27.7 %) and the IADPSG criteria (22.6 %). Thus, the current DIPSI guidelines of doing a single-step non-fasting OGTT using the 2-h VPG cut point of 140 mg/dl (7.8 mmol/l) to diagnose GDM would miss 72.3 % of women with GDM diagnosed by the WHO 1999 criteria and 77.4 % of women with GDM diagnosed by the IADPSG criteria.

Admittedly, in developing countries like India, women have to travel long distances to attend antenatal clinics. Hence, it has been felt by many obstetricians and physicians that getting all pregnant women to come in a fasting state would be a great challenge [13, 14]. Thus, performing a non-fasting OGTT emerged as a logical option and this has become very popular in India. However, given that the sensitivity of the non-fasting OGTT is low, the present report suggests that it cannot be used as a single-step definitive diagnostic test.

One of the assumptions on which the DIPSI guidelines were framed was that it is difficult to get pregnant women to come on an empty stomach for a fasting OGTT. We found that 1,071 out of 1,364 (78.5 %) pregnant women did come back for the fasting OGTT in this study, although admittedly this was in a study mode. However, it is reasonable to assume that once women are told that they are likely to have GDM based on a screening test, the compliance rates for the second definitive OGTT would improve further due to better motivation. Another presumed advantage of the non-fasting OGTT is that the frequency of women who would vomit would be higher if the glucose drink is consumed on an empty stomach. Our data show that there was no significant difference in the number of women who vomited after the fasting, compared to the non-fasting OGTT.

Based on the findings of this study, we suggest the following strategy. If a single-step screening and diagnostic test are to be used for GDM, the OGTT has to be done in the fasting state and the IADPSG or WHO 1999 criteria can be used depending on the resources available. Alternatively, if it is not possible to get all pregnant women to come back in the fasting state, the well-established two-step procedure can be continued, using the 50 g glucose challenge test (GCT) as the initial screening test [4]. Those who screen positive (i.e. 1 h ≥ 140 mg/dl) can be referred for the second step definitive OGTT done in the fasting state using either the WHO 1999 criteria (which needs only one blood sample) or the now more widely accepted IADPSG criteria which need three samples (if the OGTT is done at 24–28 weeks of gestation) depending on the resources available.

The IADPSG criteria, although adopted recently by a WHO expert group [7], may be difficult to adopt in some developing countries due to shortage of trained phlebotomists, extra costs and the lack of laboratory support. Moreover, some reports from the Western countries state that the use of IADPSG criteria could lead to inflated rates of GDM [15, 16].

Another review suggests that in low-resource settings where universal screening using a glucose challenge or an OGTT is not feasible, the use of fasting plasma glucose at 24–28 weeks may be a practical approach. In a study performed at 15 Chinese hospitals, if the OGTT was limited to women with fasting plasma glucose (FPG) ranging between 79 mg/dl (4.4 mmol/l) and 90 mg/dl (5 mmol/l), more than half of the pregnant women could avoid doing an OGTT. However, this approach may not be applicable to South Asians, who have a relatively higher prevalence of GDM. Furthermore, studies have shown that fasting plasma glucose values tend to have low sensitivity in South Asians [17, 18]. Also, as seen in the HAPO study, different sets of women were identified to have GDM by the fasting plasma glucose (8.3 %), 1 h (5.7 %) and 2 h (2.1 %). Hence, the fasting plasma glucose alone may not be reliable for diagnosis of GDM [6].

One of the limitations of this study is that maternal and foetal outcomes based on these recommendations are not available and these data are urgently needed. Secondly, the study participants were not randomized with the non-fasting and fasting tests which could have introduced a bias, but it is unlikely that this would have affected the conclusions drawn from the study.

In summary, this study demonstrates that the current DIPSI guidelines for India of adopting a single-step non-fasting OGTT using a 2-h VPG cut point of 140 mg/dl (7.8 mmol/l) as a screening and diagnostic test for GDM may need to be revisited. Ideally, and whenever feasible, a single-step 75-g OGTT using the IADPSG criteria should be done in the fasting state as this is being increasingly accepted worldwide and would help to bring about international standardization. However, in resource-limited settings, especially in the rural areas of developing countries, where getting all pregnant women to come in a fasting state may be difficult, the well-validated two-step procedure using the 50 g OGCT in the non-fasting state as the initial screening test, followed by fasting OGTT for definitive diagnosis in those who screen positive, is an adequate alternative.
